# Pathophysiological Significance of Variants of the HAND1 Gene Promoter in Congenital Atrial Septal Defects: A Study in 632 Chinese Subjects

**DOI:** 10.1155/humu/8840490

**Published:** 2026-04-30

**Authors:** Jia-Le Qi, Huan-Xin Chen, Hai-Tao Hou, Qin Yang, Guo-Wei He

**Affiliations:** ^1^ Department of Cardiovascular Surgery and the Center for Basic Medical Research, TEDA International Cardiovascular Hospital, Tianjin University, Tianjin, China, tju.edu.cn; ^2^ Tianjin Key Laboratory of Molecular Regulation of Cardiovascular Diseases and Translational Medicine, Tianjin, China; ^3^ The Institute of Cardiovascular Diseases, Tianjin University, Tianjin, China, tju.edu.cn; ^4^ TEDA International Cardiovascular Hospital, Chinese Academy of Medical Sciences, Tianjin, China, cacms.ac.cn

**Keywords:** atrial septal defect, congenital heart disease, genetic, HAND1

## Abstract

**Background:**

Atrial septal defect (ASD) is a common congenital heart disease (CHD) and genetic variation in the HAND1 gene is associated with cardiac development. The variants in the promoter region of the HAND1 gene are unknown.

**Methods:**

We performed Sanger sequencing of DNA from 632 subjects (320 ASD patients and 312 healthy controls). The identified variants were also subjected to further cellular functional validation, electrophoretic mobility shift analysis (EMSA), and JASPAR database analysis.

**Results:**

A total of 12 variants were identified in the promoter region of HAND1 gene, seven of which were found only in 10 ASD patients (g.3658 T > C [rs1287904093], g.3689 A > G, g.3714 G > A [rs140545341], g.3771 C > T [rs2113306555], g.3961 T > G, g.4411 A > T [rs1034236730], and g.4512 G > T) and three of the variants (g.3689 A > G, g.3961 T > G, and g.4512 G > T) were newly discovered. Further cellular functional validation showed that these seven variants reduced the transcriptional activity of HAND1 gene promoter (p < 0.05). The results of EMSA and the analysis of the JASPAR database suggest that these variants may have altered a series of transcription factor binding sites (TFBs), leading to altered HAND1 protein expression as well as the development of ASD.

**Conclusions:**

Thus, the present study provides new insights into the role of the promoter region of HAND1 gene, which could lead to a better understanding of the genetic basis of ASD formation and potential treatments.

## 1. Introduction

Congenital heart disease (CHD) is the most common of all congenital anomalies, accounting for approximately 28% of all births [1], and is a major global health problem. Compared with other birth defects, CHD is a significant cause of human neonatal morbidity and mortality, with prevalence rates ranging from 4/1000 live births to 10/1000 [2]. Moreover, defects in the cardiac conduction system significantly increase the risk of death [3]. CHD can be broadly categorized into two main groups: (1) morphologic abnormalities, which include developmental defects that result in structural malformations; and (2) functional abnormalities, which include cardiac rhythm disorders and cardiomyopathies [4]. Although the genetic mechanisms underlying many CHD are currently unknown, variants in genes encoding core cardiac transcription factors (TFs) have emerged as a major causative factor in a variety of CHD [4].

Atrial septal defect (ASD) is a common CHD characterized by insufficient or absent septal tissue [5]. ASD affects 1 in 1500 live births and accounts for 6%–10% of CHD [6]. Adults with ASD have about a 10% risk of recurrent heart disease in their offspring [7]. There are three main types of ASD: secondary foramen ovale, primary foramen ovale, and venous sinus [8]. The pathogenesis of ASD is heavily influenced by genetic defects, but the genetic determinants of ASD are unknown [9].

The name Hand is derived from the tissues in which proteins are expressed at the embryonic stage: the heart, the autonomic nervous system, and neural peak derivatives [10]. The basic helix‐loop‐helix (bHLH) factors HAND1 and HAND2 are determinants of right and left ventricular formation [11] and regulate ventricular precursor dilation in a dose‐dependent manner [12]. Ventricle‐specific analysis of HAND gene expression has shown that human HAND2 is expressed in all ventricles of the normal adult heart, whereas HAND1 expression is relatively ventricle‐specific [13]. For mice deficient in the HAND1 gene, cardiac development is arrested at the annular stage of cardiac development [14]. Conditionally activated HAND1 knock‐in mice have increased epicardial dilatation of both ventricles but lack the interventricular sulcus and have defective septal formation [15].

Human genetics studies have identified approximately 400 genes involved in the pathogenesis of inherited and sporadic CHD [16]. These include TFs, cell signaling molecules, and structural proteins that are essential for heart development. If these genes are mutated, they can interfere with processes such as cell differentiation during cardiac development, leading to structural and functional disorders of the heart [15]. Based on our previous studies of the promoter regions of other genes associated with cardiac development in patients with CHD, such as CITED2 [17], ISL1 [18] and MYH6 [19–21], we hypothesized that variants in the promoter region of the HAND1 gene might also affect ASD formation. To test this hypothesis, we screened for variants in the promoter region of the HAND1 gene in ASD patients and healthy controls. In addition, we validated it at the cellular functional level for further analysis.

## 2. Materials and Methods

### 2.1. Subjects of Study

**Figure 1:**
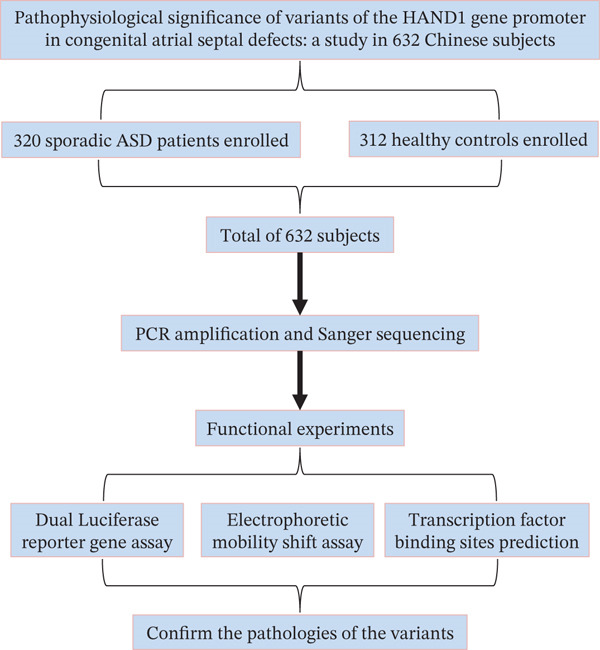
Flow chart of the experiment. A total of 632 subjects participated in this study, including 320 ASD patients and 312 healthy controls. Sequence analysis, cellular function experiments, electrophoretic migration analysis, and bioinformatics analysis were subsequently performed. ASD: atrial septal defect.

In this study, blood samples were collected from 632 subjects enrolled in the study, including 320 patients with isolated ASD and 312 healthy controls. Exclusion criteria were patients with no blood samples, a family history of CHD, or comorbidities of other genetic disorders. We defined these patients as “sporadic ASD patients”. All patients underwent corrective surgery at the TEDA International Cardiovascular Hospital, Tianjin University. There were no significant differences between healthy controls and patients in terms of ethnicity or gender. The study was approved by the Ethics Committee of TEDA International Cardiovascular Hospital (Clinical Research Ethics Review No: [2021]‐0715‐4), Tianjin University. The parents or guardians of all subjects signed the informed consent form. In addition, this study strictly followed the guidelines of the Declaration of Helsinki. Figure 1 illustrates the experimental flow of this study.

### 2.2. DNA Sequence Analysis of the Promoter Region of HAND1 Gene

Peripheral blood leukocytes were isolated from blood specimens using a blood genome kit, and polymerase chain reaction (PCR) primers were designed with reference to the promoter sequence of HAND1 gene in the GenBank database (NCBI, NG_052889.1) (Table 1). The suitable PCR system and conditions were set (predenaturation at 95°C for 5 min, denaturation at 95°C for 30 s, annealing at 60°C for 30 s, extension at 72°C for 105 s for a total of 35 cycles, and final extension for 3 min). The obtained amplification products were subjected to Sanger bidirectional sequencing. The obtained sequencing results were performed with the wild‐type sequence of HAND1 gene promoter to screen for variants.

**Table 1:** The primer list used in this study.

Primers name	Sequences	Location
**PCR primers**		
HAND1pro‐F_3_	5 ^′^‐ATCCACTCCCTACCGGACC‐3 ^′^	3371–3389
HAND1pro‐R_3_	5 ^′^‐GCCGCCAGCCCTATTAACG‐3 ^′^	5216–5234

**Sequencing primers**		
HAND1pro‐F_3_	5 ^′^‐ATCCACTCCCTACCGGACC‐3 ^′^	3371–3389
HAND1pro‐R_3_	5 ^′^‐GCCGCCAGCCCTATTAACG‐3 ^′^	5216–5234

**The double-stranded biotinylated oligonucleotides for the EMSA**		
g.3658 T > C‐F	5 ^′^‐TTGAGCTGAGAAAGGT(C)TGAGAGAATGAGGGA‐3 ^′^	
g.3658 T > C‐R	5 ^′^‐TCCCTCATTCTCTCAA(G)CCTTTCTCAGCTCAA‐3 ^′^	
g.3689A > G‐F	5 ^′^‐CCCAGGTAGGTGGACA(G)TCGGCCAAGAAAGGA‐3 ^′^	
g.3689A > G‐R	5 ^′^‐TCCTTTCTTGGCCGAT(C)GTCCACCTACCTGGG‐3 ^′^	
g.3714G > A‐F	5 ^′^‐AAAGGAACCACAGCGG(A)GAGGTAAGACCGAGA‐3 ^′^	
g.3714G > A‐R	5 ^′^‐TCTCGGTCTTACCTCC(T)CGCTGTGGTTCCTTT‐3 ^′^	
g.3771C > T‐F	5 ^′^‐CGGGATTCCCAGATTC(T)CAACGCGAGCCTGGG‐3 ^′^	
g.3771C > T‐R	5 ^′^‐CCCAGGCTCGCGTTGG(A)AATCTGGGAATCCCG‐3 ^′^	
g.3961 T > G‐F	5 ^′^‐TTGGTCCCCTCTGCGT(G)GTGTCTAACGCCGAA‐3 ^′^	
g.3961 T > G‐R	5 ^′^‐TTCGGCGTTAGACACA(C)CGCAGAGGGGACCAA‐3 ^′^	
g.4411A > T‐F	5 ^′^‐GCACAAGCGGCTCCCA(T)GTCTCTCCAGAAAGG‐3 ^′^	
g.4411A > T‐R	5 ^′^‐CCTTTCTGGAGAGACT(A)GGGAGCCGCTTGTGC‐3 ^′^	
g.4512G > T‐F	5 ^′^‐CCTGTCTTCAGCAGCG(T)CCCTCTCATCTTCTA‐3 ^′^	
g.4512G > T‐R	5 ^′^‐TAGAAGATGAGAGGGC(A)GCTGCTGAAGACAGG‐3 ^′^	

Note: According to the genomic DNA sequence of HAND1 gene (NG_052889.1), design PCR primers. The transcription start site is at the position of 5033(+1) [29].

Abbreviations: EMSA, electrophoretic mobility shift assay; F, forward; R, reverse.

### 2.3. Plasmid Construction and Cell Transfection

In order to study the effect of variant on promoter activity, the expression vector was first constructed by cloning the wild‐type fragment into the KpnI/HindIII site of pGL3‐Basic (pGL3‐Basic is a reporter gene vector expressed by firefly luciferase) by restriction endonuclease digestion. In addition, the vector sequence was verified by Sanger sequencing.

Plasmids carrying the target genes were transformed into DH5*α* cells. Similarly, bacterial fluids containing variants pGL3‐V3658, pGL3‐V3689, pGL3‐V3714, pGL3‐V3771, pGL3‐V3961, pGL3‐V4411, and pGL3‐V4512 were obtained and stored in a −80°C refrigerator for spare use. Plasmids were extracted using fresh bacterial fluids.

HL‐1 cells are an atrial myocyte cell line derived from AT‐1 mouse atrial tissue and exhibit genomic characteristics similar to those of humans. HL‐1 cells were resuscitated and cultured in complete MEM/DMEM (10% fetal bovine serum +1% penicillin and streptomycin). One day before transfection, 0.25 − 1 × 10^6^ cells were cultured into 6‐well plates. When the cells grew to 60%–80%, the expression plasmid was cotransfected into HL‐1 cells with the renilla luciferase reporter plasmid pRL‐SV40. Sea kidney luciferase reporter plasmid (pRL‐SV40) was used as an internal control. The cells were continued to be cultured in six‐well culture plates for 24–48 h, and the cell morphology was observed at any time.

### 2.4. Dual‐Luciferase Reporter Assay

The transfected cells were collected and fully lysed, and an equal volume of cell lysate was taken as a blank control. Firefly luciferase and renilla luciferase activities were measured according to the dual‐luciferase reporter assay system (Beyotime Biotechnology, Beijing, China). The transcriptional activity of HAND1 gene was assessed by its relative value. The experiments were performed three times independently, and three groups were repeated each time.

### 2.5. Cell Nuclear Extract Preparation and Electrophoretic Mobility Shift Analysis (EMSA)

Cytosolic proteins were extracted with the cytosolic protein and cytoplasmic protein extraction kit (Beyotime, China) and stored in a −80°C refrigerator for backup. Biotinylated double‐stranded oligonucleotides with wild and variant‐type sequences of HAND1 gene promoter were designed as probes (Table 1). EMSA was performed using a chemiluminescent EMSA kit (Beyotime, China), and gel imaging results were obtained.

### 2.6. Transcription Factor Binding Sites (TFBs) Prediction

To predict whether the identified variants would affect the TFBs in the HAND1 promoter region, the JASPAR database was used to predict all the TFBs that could be disrupted or generated by the variants [22]. The relative threshold was set to 85%.

### 2.7. Statistical Analysis

All statistical analyses were performed using SPSS 26.0. The quantitative data were also compared using the standard Student t‐test. p < 0.05 was considered statistically significant.

## 3. Results

### 3.1. Variants in the Promoter Region of HAND1 Gene in ASD Patients and Healthy Controls

**Figure 2:**
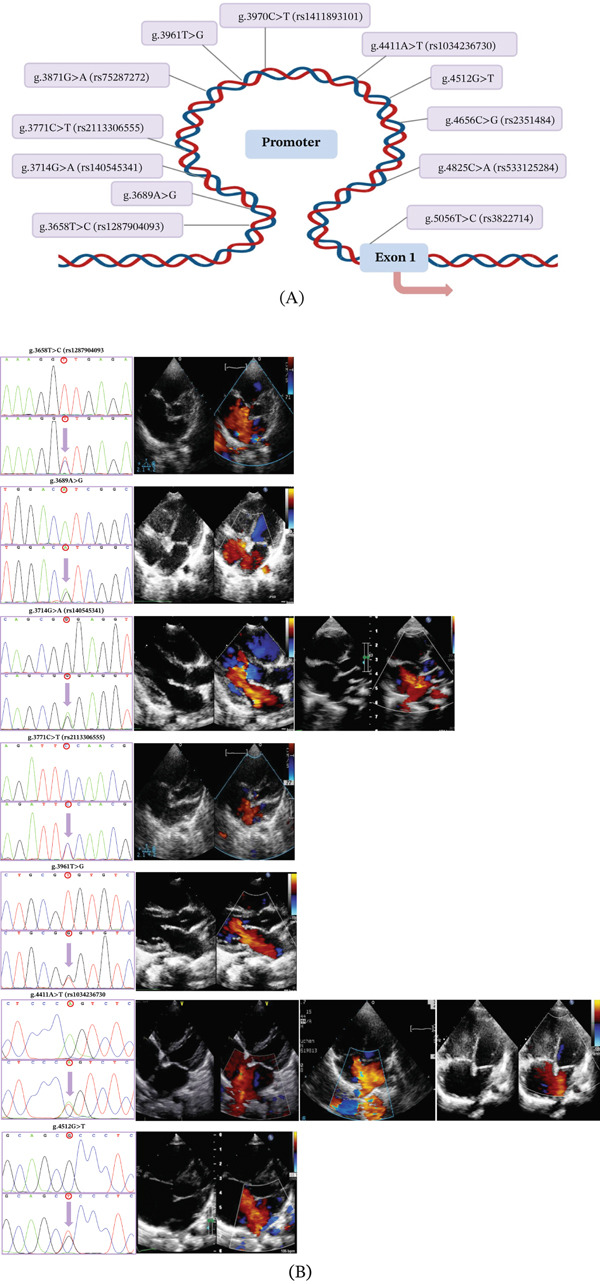
Location of variants in HAND1 gene promoter, sequencing chromatograms, and Doppler echocardiography. (A) According to the genomic DNA sequence of HAND1 gene (NG_052889.1), the variants are named according to the point of variant, and the transcription start site is located at position 5033 in the first exon. The locations of the 12 promoter variants in the HAND1 gene are shown. (B) Left: Seven variants found only in ASD patients (g.3658 T > C (rs1287904093); g.3689 A > G; g.3714 G > A (rs140545341); g.3771 C > T (rs2113306555); g.3961 T > G; g.4411 A > T (rs1034236730); and g.4512 G > T) sequencing chromatograms. The upper panel indicates the wild type and the lower panel indicates the variant type with arrows. Right: Color Doppler echocardiography revealed that the 10 patients carrying the variants had large ASDs.

In this study, Sanger sequencing was performed on 632 Chinese subjects (320 ASD patients and 312 healthy controls), and 12 variants were identified (Table 2). In addition, the locations of the variants are shown in Figure 2A, and the sequencing profiles are shown on the left side of Figure 2B. Among these 12 variants, seven of them (g.3658 T > C [rs1287904093]; g.3689A > G; g.3714G > A [rs140545341]; g.3771C > T [rs2113306555]; g.3961 T > G; g.4411A > T [rs1034236730]; g.4512G > T) were found only in ASD patients, whereas the other five variants were found in both ASD patients and healthy controls. Importantly, three of these variants (g.3689A > G; g.3961 T > G; and g.4512G > T) were found for the first time and have not been reported previously (HAND1—SNP—NCBI https://www.ncbi.nlm.nih.gov/snp/?term=HAND1). Secondly, variant g.3714G > A (rs140545341) was found in two ASD patients and variant g.4411A > T (rs1034236730) in three ASD patients. In addition, the allele frequencies of these seven variants found only in ASD patients were all < 0.0001 in the NCBI SNP database, suggesting that they may be potentially pathogenic. Therefore, these seven variants were further investigated in cellular functional validation. In contrast, the other five variants found in both ASD patients and healthy controls were excluded from further study. Figure 2B on the right shows color Doppler echocardiograms of 10 patients carrying the variants, revealing that these patients have large ASDs. Among these 10 patients with ASD, there were four males and six females with the age of 6.2 ± 4.7 years (mean ± SD; ranged: 0.85–13 years with one of 15 years).

**Table 2:** Variants in HAND1 gene promoter region of ASD patients and healthy controls.

Variants	ASD	Controls	Position ^∗^	Allele frequency							
**Frequency in control = 0 (further validation)**				**ALFA** ^**a**^		**1000 Genomes** ^**b**^		**1000 Genomes_30x** ^**c**^		**gnomAD–genomes** ^**d**^	
				**East-Asian**	**Global**	**East-Asian**	**Global**	**East-Asian**	**Global**	**East-Asian**	**Global**
g.3658 T > C (rs1287904093)	1	0	−1376	G = 0.00	G = 0.00000	None	None	None	None	None	None
g.3689A > G	1	0	−1344	None	None	None	None	None	None	None	None
g.3714G > A (rs140545341)	2	0	−1319	T = 0.000	T = 0.00619	T = 0.0000	T = 0.0038	T = 0.0000	T = 0.0036	None	None
g.3771C > T (rs2113306555)	1	0	−1262	None	None	None	None	None	None	None	None
g.3961 T > G	1	0	−1072	None	None	None	None	None	None	None	None
g.4411A > T (rs1034236730)	3	0	−622	A = 0.00	A = 0.00000	None	None	None	None	A = 0.0010	A = 0.000029
g.4512G > T	1	0	−521	None	None	None	None	None	None	None	None

**Frequency in control ≠ 0 (no further validation)**											
g.3871G > A (rs75287272)	14	5	−1162	T = 0.02	T = 0.00014	T = 0.0218	T = 0.0046	T = 0.0214	T = 0.0039	T = 0.0252	T = 0.000592
g.3970C > T (rs1411893101)	2	1	−1063	A = 0.00	A = 0.00000	None	None	None	None	None	None
g.4656C > G (rs2351484)	131	125	−377	C = 0.38	C = 0.18883	C = 0.2976	C = 0.2067	C = 0.2949	C = 0.2038	C = 0.2804	C = 0.174987
g.4825C > A (rs533125284)	1	1	−208	T = 0.00	T = 0.00000	None	None	None	None	None	None
g.5056 T > C (rs3822714)	105	78	+24	G = 0.03	G = 0.07615	G = 0.2798	G = 0.1428	G = 0.2812	G = 0.1390	None	None

Note: The asterisk ( ^∗^) denotes the position of variants are relative to the transcription start site at 5033 (+1) [29] of HAND1 gene (NG_052889.1).

^a^ALFA allele frequency, the ALFA project provide aggregate allele frequency from dbGaP (20230706150541).

^b^1000 genomes project phase3 release V3+, this submission contains variant sites and phased genotypes for all autosomes (excluding a 3Mbps region on Chromosome 12 between 7 M and 10 M) derived from the 1000 genomes project phase3 sequence data (PRJEB6930).

^c^1000 genomes project Phase 3: 30X whole‐genome sequencing coverage of the 2504 phase 3 1000 genome samples. (PRJEB31736).

^d^The genome aggregation database (gnomAD), the data set provided on this website spans 123,136 exome sequences and 15,496 whole‐genome sequences from unrelated individuals sequenced as part of various disease‐specific and population genetic studies (PRJNA398795).

### 3.2. Dual‐Luciferase Reporter Assay Results

To further determine whether HAND1 gene promoter region variants affect transcriptional activity, we constructed different reporter gene expression vectors based on HAND1 gene promoter region fragments. These included pGL3‐Basic, pGL3‐WT, pGL3‐V3658, pGL3‐V3689, pGL3‐V3714, pGL3‐V3771, pGL3‐V3961, pGL3‐V4411, and pGL3‐V4512. These constructed vector plasmids were then transfected into HL‐1 cells, respectively. A dual‐luciferase reporter assay was subsequently performed.

**Figure 3:**
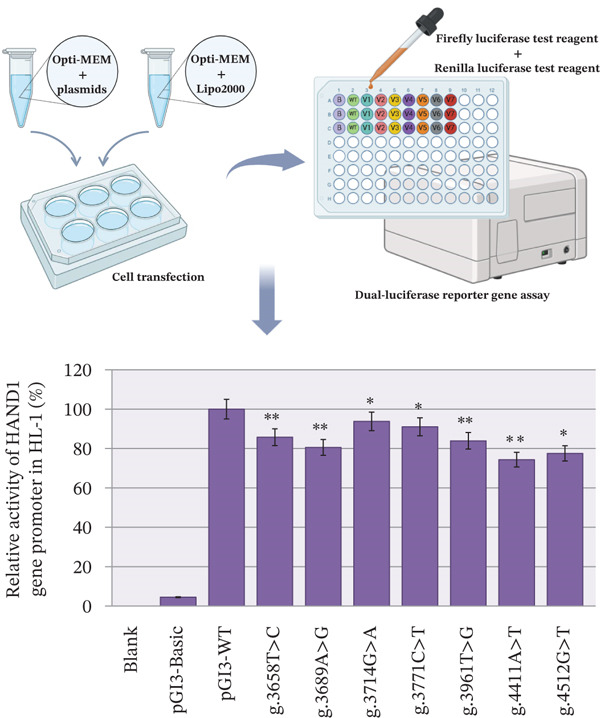
Results of dual luciferase reporter gene assay. Relative transcriptional activity data for wild and variant‐types HAND1 gene promoters in HL‐1 cells are shown and expressed as mean ± SD. Experiments were performed three times with three replicates each (∗p < 0.05, ∗∗p < 0.01, ∗∗∗p < 0.001).

As shown in Figure 3, the transcriptional activities of the blank and negative control groups were significantly lower than the other seven groups. Importantly, all seven variants significantly reduced HAND1 gene promoter expression activity as well as dual luciferase expression (p < 0.05).

### 3.3. EMSA Results

**Figure 4:**
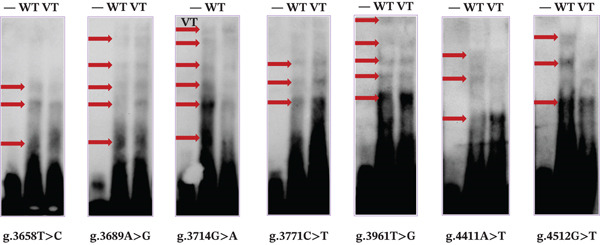
EMSA results. Biotin probes were analyzed by EMSA with seven different variants of wild and variant types, respectively. It was found that seven variants in cells (g.3658 T > C (rs1287904093); g.3689 A > G; g.3714 G > A (rs140545341); g.3771 C > T (rs2113306555); g.3961 T > G; g.4411 A > T (rs1034236730); and g.4512 G > T) can enhance or disrupt transcription factor binding. EMSA: electrophoretic mobility shift analysis; WT: wild type; VT: variant type.

To verify whether the variants affect TFBs, we performed EMSA analysis of seven variants found only in ASD patients using wild and variant biotin‐labeled probes. The biotinylated oligonucleotide sequences of EMSA are shown in Table 1. The brightness of the bands reflects the TFs′ binding capacity [23], and arrows indicate bands of different brightness. As shown in the results of Figure 4, compared with the wild type, the variants g.3658 T > C (rs1287904093); g.3689 A > G; g.3714 G > A (rs140545341); g.3771 C > T (rs2113306555); g.3961 T > G; g.4411 A > T (rs1034236730), and g.4512 G > T all significantly enhanced or eliminated the binding ability of TFs.

### 3.4. Predicting the Effect of Variants in the Promoter Region of HAND1 Gene on TFBs

We predicted the promoter region of HAND1 gene using the JASPAR database to further investigate whether the identified variants affect the TFBs. As shown in Table 3, variant g.3658 T > C (rs1287904093) produced three binding sites, including FEZF1, and disrupted six binding sites, including NR4A2; variant g.3689 A > G produced five binding sites, including HINFP, and disrupted three binding sites, including MEIS1; variant g.3714 G > A (rs140545341) disrupts 13 binding sites including E2F1; variant g.3771 C > T (rs2113306555) produces six binding sites including GATA3 and disrupts eight binding sites including NFIC; variant g.3961 T > G produces eight binding sites including GCM1 and disrupts eight ARNT::HIF1A binding sites; variant g.4411 A > T (rs1034236730) produced six binding sites including CTCFL and disrupted six binding sites including FEZF2; variant g.4512 G > T produced eight binding sites including E2F6 and disrupted 12 binding sites including CTCFL.

**Table 3:** TFBs predicted by the JASPAR database.

Variations	Binding sites for transcription factors	
	Create	Disrupt
g.3658 T > C (rs1287904093)	FEZF1, FEZF2, ZNF264	NR4A2, NR2C2, NKX2‐5, NR1I3, SNAI2, ZNF627
g.3689A > G	HINFP, HIF1A, ZBTB7A, ZBTB7C, ZBTB21	MEIS1, MEIS3, GATA2
g.3714G > A (rs140545341)	\	E2F1, E2F4, E2F6, ZIC3, ZIC4, ZIC5, ZNF263, ZNF462, ZBTB21, FOXC1, THAP1, TFDP1, YBX1
g.3771C > T (rs2113306555)	GATA3, GATA5, NR2C2, REL, SOX18, TWIST1	NFIC, NFIX, NFATC4, TEAD1, TEAD2, TEAD3, TEAD4, ETS1
g.3961 T > G	GCM1, GCM2, RUNX2, RUNX3, TBX3, TCF3, SNAI1, ZEB1	ARNT::HIF1A, DMRTA2, FOXL1, HES1, MXI1, NEUROD2, TCF15, TCFL5
g.4411A > T (rs1034236730)	CTCFL, MEIS1, MEIS3, PRDM1, ZNF781, ZNF823	FEZF2, ZNF222, ZNF337, ZNF611, ZNF736, ZNF786
g.4512G > T	E2F6, NRL, NHLH1, ZNF483, ZNF519, ZNF594, ZNF781, ZNF860	CTCFL, HIC2, KLF2, KLF3, NR2C1, NR2C2, RFX7, ZNF30, ZNF425, ZNF548, ZNF554, ZNF783

**Figure 5:**
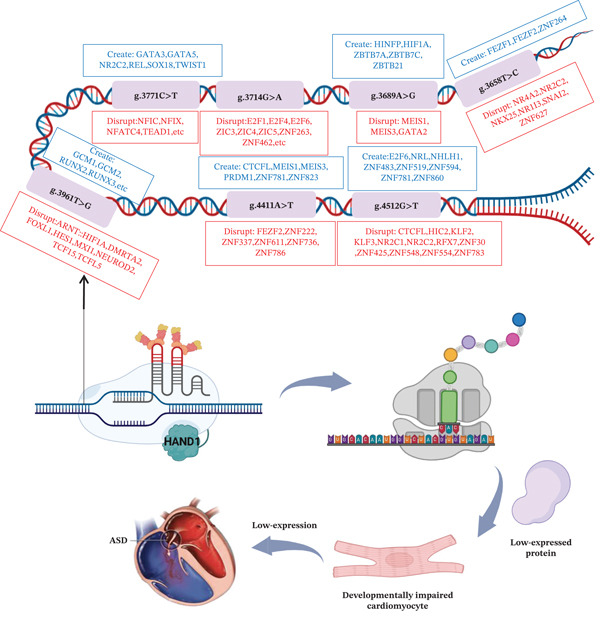
Effect of variants in the promoter region of HAND1 gene on certain TFs and cardiac development. ASD: atrial septal defect.

Based on the results of this study in cell function validation, EMSA analysis, and JASPAR database prediction, Figure 5 illustrates the effects of HAND1 promoter region variants on a variety of TFs and their possible impact on cardiac development.

## 4. Discussion

In this study, we performed the first genetic and functional analysis of the promoter region of the HAND1 gene in patients with isolated ASD versus healthy controls.

A total of 12 variants were identified in 632 subjects, seven of which (g.3658 T > C (rs1287904093); g.3689 A > G; g.3714 G > A (rs140545341); g.3771 C > T (rs2113306555); g.3961 T > G; g.4411 A > T (rs1034236730); g.4512 G > T) were found only in ASD patients. These seven variants had low allele frequencies in the total and East Asian populations in the NCBI database. More importantly, three variants (g.3689 A > G; g.3961 T > G; g.4512 G > T) have never been previously reported. Variant g.3714 G > A (rs140545341) was found in two patients with ASD and variant g.4411 A > T (rs1034236730) was found in three patients. In addition, dual luciferase reporter gene assays demonstrated that all seven variants resulted in a significant reduction in the transcriptional activity of HAND1 gene promoter. EMSA and bioinformatics analyses showed that all seven variants affect TFBs. It was hypothesized that variants in the promoter region of HAND1 gene may be involved in the formation of ASD.

Based on the analysis of the results in Table 3, both HAND1 and HAND2, which are key genes for heart development, can activate the NPPA promoter together with MEF2 [24, 25], whereas for the trans activation of HAND1, only the MEF2 binding site in the DNA is required [26]. The binding of HAND1 did not increase the DNA binding of MEF2, so it is believed that the increase in promoter activity is obtained through the additional factor of attracting the promoter by HAND1 [25]. In addition to this, HAND1 and HAND2 can form homodimers or heterodimers with themselves and other Class B bHLH factors [26].

TFs in CHD patients have been found to contain de novo and loss‐of‐function mutations [27]. Proteins with such deleterious mutations may have altered transcriptional or synergistic activities, which may interfere with the expression of downstream targets and affect normal cell development and differentiation [28].

As shown in Figure 5, we summarize the relevant mechanisms by which variants in the promoter region of HAND1 gene may occur in ASD. In the present study, the described variants all resulted in reduced HAND1 gene promoter activity, which in turn led to altered cellular function. Variants in the promoter region of HAND1 gene may reduce the expression of genes that are essential for cardiac morphogenesis during cardiac development, thereby affecting cardiac development and contributing to the genetic susceptibility of ASD.

### 4.1. Limitations

We may find more variants in the promoter region of the HAND1 gene if they are studied in a larger population of ASD patients. In addition, in vivo studies in further animal models may better confirm the role of HAND1 gene promoter variants in CHD development.

## 5. Conclusion

In conclusion, our study identifies for the first time several variants in the promoter region of the HAND1 gene in Chinese patients with isolated and sporadic ASD. In addition, these variants may contribute to the development of ASD by affecting the expression of the HAND1 gene and altering the TFBs. This study provides new ideas to further understand the genetic basis and molecular mechanisms of CHD formation.

## Author Contributions

Writing—original draft: Jia‐Le Qi. Investigation: Jia‐Le Qi. Methodology: Jia‐Le Qi, Huan‐Xin Chen, Hai‐Tao Hou, Guo‐Wei He. Data curation: Jia‐Le Qi, Huan‐Xin Chen, Hai‐Tao Hou, Qin Yang, Guo‐Wei He. Validation: Jia‐Le Qi, Guo‐Wei He. Formal analysis: Jia‐Le Qi, Hai‐Tao Hou, Guo‐Wei He. Conceptualization: Guo‐Wei He. Writing—review and editing: Huan‐Xin Chen, Guo‐Wei He. Supervision, visualization, resources, project administration, and funding acquisition: Guo‐Wei He.

## Funding

This study was supported by National Natural Science Foundation of China (82370350 and 82170353); TEDA International Cardiovascular Hospital (2021‐ZX‐002); Tianjin Key Medical Discipline Construction Project (TJYXZDXK‐3‐036C); Special Fund for High Quality Development Project.

## Consent

All subjects participating in the study have obtained written informed consent from their parents or guardians.

## Conflicts of Interest

The authors declare no conflicts of interest.

## Data Availability

The individual SNP numbers are given in Table 2. The genetic variants described in this manuscript are available at https://www.ncbi.nlm.nih.gov/snp/. Data supporting the findings of this study are available upon reasonable request from the corresponding author.

## References

[1] Dolk H. , Loane M. , and Garne E. , Congenital Heart Defects in Europe: Prevalence and Perinatal Mortality, 2000 to 2005, Circulation. (2011) 123, no. 8, 841–849, 10.1161/CIRCULATIONAHA.110.958405, 2-s2.0-79952440734. 21321151

[2] Miranovic V. , The Incidence of Congenital Heart Disease: Previous Findings and Perspectives, Srpski Arhiv Za Celokupno Lekarstvo. (2014) 142, no. 3-4, 243–248, 10.2298/SARH1404243M, 2-s2.0-84940345325, 24839784. 24839784

[3] Hoffman J. I. , Kaplan S. , and Liberthson R. R. , Prevalence of Congenital Heart Disease, Am Heart J.(2004) 147, 425–439. 14999190 10.1016/j.ahj.2003.05.003

[4] McCulley D. J. and Black B. L. , Transcription Factor Pathways and Congenital Heart Disease, Current Topics in Developmental Biology. (2012) 100, 253–277, 10.1016/B978-0-12-387786-4.00008-7, 2-s2.0-84858770543, 22449847. 22449847 PMC3684448

[5] Bradley E. A. and Zaidi A. N. , Atrial Septal Defect, Cardiology Clinics. (2020) 38, no. 3, 317–324, 10.1016/j.ccl.2020.04.001. 32622487

[6] Haas N. A. , Driscoll D. J. , and Rickert-Sperling S. , Clinical Presentation and Therapy of Atrial Septal Defect, Advances in Experimental Medicine and Biology. (2024) 1441, 461–466, 10.1007/978-3-031-44087-8_23. 38884725

[7] Uebing A. , Steer P. J. , Yentis S. M. , and Gatzoulis M. A. , Pregnancy and Congenital Heart Disease, British Medical Journal. (2006) 332, no. 7538, 401–406, 10.1136/bmj.38756.482882.DE. 16484266 PMC1370974

[8] Webb G. and Gatzoulis M. A. , Atrial Septal Defects in the Adult, Circulation. (2006) 114, no. 15, 1645–1653, 10.1161/CIRCULATIONAHA.105.592055, 2-s2.0-33749621392. 17030704

[9] Chen H. X. , Hou H. T. , Wang X. L. , Wang J. , Yang Q. , and He G. W. , Functional FOXC1 Variants in Familial and Sporadic Atrial Septal Defect With Cellular and Animal Validation, Clinical and Translational Medicine. (2024) 14, no. 7, e1676, 10.1002/ctm2.1676, 38924312. 38924312 PMC11199059

[10] Cserjesi P. , Brown D. , Lyons G. E. , and Olson E. N. , Expression of the Novel Basic Helix-Loop-Helix Gene eHAND in Neural Crest Derivatives and Extraembryonic Membranes During Mouse Development, Developmental Biology. (1995) 170, no. 2, 664–678, 10.1006/dbio.1995.1245, 2-s2.0-0029142976, 7649392. 7649392

[11] Thomas T. , Yamagishi H. , Overbeek P. A. , Olson E. N. , and Srivastava D. , The bHLH factors, dHAND and eHAND, Specify Pulmonary and Systemic Cardiac Ventricles Independent of Left-Right Sidedness, Developmental Biology. (1998) 196, no. 2, 228–236, 10.1006/dbio.1998.8849, 2-s2.0-0032523101, 9576835. 9576835

[12] McFadden D. G. , Barbosa A. C. , Richardson J. A. , Schneider M. D. , Srivastava D. , and Olson E. N. , The Hand1 and Hand2 Transcription Factors Regulate Expansion of the Embryonic Cardiac Ventricles in a Gene Dosage-Dependent Manner, Development. (2005) 132, no. 1, 189–201, 10.1242/dev.01562, 2-s2.0-13444310919, 15576406. 15576406

[13] Natarajan A. , Yamagishi H. , Ahmad F. , Li D. , Roberts R. , Matsuoka R. , Hill S. , and Srivastava D. , Human eHAND, but not dHAND, Is Down-Regulated in Cardiomyopathies, Journal of Molecular and Cellular Cardiology. (2001) 33, no. 9, 1607–1614, 10.1006/jmcc.2001.1434, 2-s2.0-0034844133. 11549340

[14] Firulli B. A. , Toolan K. P. , Harkin J. , Millar H. , Pineda S. , and Firulli A. B. , The HAND1 Frameshift A126FS Mutation Does Not Cause Hypoplastic Left Heart Syndrome in Mice, Cardiovascular Research. (2017) 113, no. 14, 1732–1742, 10.1093/cvr/cvx166, 2-s2.0-85040096876, 29016838. 29016838 PMC5852545

[15] Williams K. , Carson J. , and Lo C. , Genetics of Congenital Heart Disease, Biomolecules. (2019) 9, no. 12, 10.3390/biom9120879. PMC6995556 31888141

[16] Bruneau B. G. , The Developmental Genetics of Congenital Heart Disease, Nature. (2008) 451, no. 7181, 943–948, 10.1038/nature06801, 2-s2.0-39749191367. 18288184

[17] Chen Z. , Chen H. X. , Hou H. T. , Yin X. Y. , Yang Q. , Han J. , and He G. W. , Genetic Variants of CITED2 Gene Promoter in Human Atrial Septal Defects Case-Control Study and Cellular Functional Verification, Journal of Cardiovascular Development and Disease. (2022) 9, no. 10, 10.3390/jcdd9100321. PMC9604052 36286273

[18] Yin X. Y. , Chen H. X. , Chen Z. , Yang Q. , Han J. , and He G. W. , Identification and Functional Analysis of Genetic Variants of ISL1 Gene Promoter in Human Atrial Septal Defects, Journal of Gene Medicine. (2022) 24, no. 12, e3450, 10.1002/jgm.3450, 36170181. 36170181

[19] Zuo J. Y. , Chen H. X. , Liu Z. G. , Yang Q. , and He G. W. , Identification and Functional Analysis of Variants of MYH6 Gene Promoter in Isolated Ventricular Septal Defects, BMC Med Genomics. (2022) 15, no. 1, 10.1186/s12920-022-01365-y. PMC9548206 36209093

[20] Zuo J. Y. , Chen H. X. , Yang Q. , Liu Z. G. , and He G. W. , Tetralogy of Fallot: Variants of MYH6 Gene Promoter and Cellular Functional Analyses, Pediatric Research. (2024) 96, no. 2, 338–346, 10.1038/s41390-023-02955-x, 38135727. 38135727

[21] Zuo J. Y. , Chen H. X. , Yang Q. , and He G. W. , Variants of the Promoter of MYH6 Gene in Congenital Isolated and Sporadic Patent Ductus Arteriosus: Case-Control Study and Cellular Functional Analyses, Human Molecular Genetics. (2024) 33, no. 10, 884–893, 10.1093/hmg/ddae021, 38340456. 38340456

[22] Castro-Mondragon J. A. , Riudavets-Puig R. , Rauluseviciute I. , Berhanu Lemma R. , Turchi L. , Blanc-Mathieu R. , Lucas J. , Boddie P. , Khan A. , Manosalva Pérez N. , Fornes O. , Leung T. Y. , Aguirre A. , Hammal F. , Schmelter D. , Baranasic D. , Ballester B. , Sandelin A. , Lenhard B. , Vandepoele K. , Wasserman W. W. , Parcy F. , and Mathelier A. , JASPAR 2022: the 9th Release of the Open-Access Database of Transcription Factor Binding Profiles, Nucleic Acids Research. (2022) 50, no. D1, D165–D173, 10.1093/nar/gkab1113, 34850907. 34850907 PMC8728201

[23] Hellman L. M. and Fried M. G. , Electrophoretic Mobility Shift Assay (EMSA) for Detecting Protein-Nucleic Acid Interactions, Nature Protocols. (2007) 2, no. 8, 1849–1861, 10.1038/nprot.2007.249, 2-s2.0-34548169696, 17703195. 17703195 PMC2757439

[24] Zang M. X. , Li Y. , Xue L. X. , Jia H. T. , and Jing H. , Cooperative Activation of Atrial Naturetic Peptide Promoter by dHAND and MEF2C, Journal of Cellular Biochemistry. (2004) 93, no. 6, 1255–1266, 10.1002/jcb.20225, 2-s2.0-11144236620, 15486975. 15486975

[25] Morin S. , Pozzulo G. , Robitaille L. , Cross J. , and Nemer M. , MEF2-Dependent Recruitment of the HAND1 Transcription Factor Results in Synergistic Activation of Target Promoters, Journal of Biological Chemistry. (2005) 280, no. 37, 32272–32278, 10.1074/jbc.M507640200, 2-s2.0-25444493136. 16043483

[26] McFadden D. G. , McAnally J. , Richardson J. A. , Charité J. , and Olson E. N. , Misexpression of dHAND Induces Ectopic Digits in the Developing Limb Bud in the Absence of Direct DNA Binding, Development. (2002) 129, no. 13, 3077–3088, 10.1242/dev.129.13.3077, 12070084. 12070084

[27] Jin S. C. , Homsy J. , Zaidi S. , Lu Q. , Morton S. , DePalma S. R. , Zeng X. , Qi H. , Chang W. , Sierant M. C. , Hung W. C. , Haider S. , Zhang J. , Knight J. , Bjornson R. D. , Castaldi C. , Tikhonoa I. R. , Bilguvar K. , Mane S. M. , Sanders S. J. , Mital S. , Russell M. W. , Gaynor J. W. , Deanfield J. , Giardini A. , Porter G. A. , Srivastava D. , Lo C. W. , Shen Y. , Watkins W. S. , Yandell M. , Yost H. J. , Tristani-Firouzi M. , Newburger J. W. , Roberts A. E. , Kim R. , Zhao H. , Kaltman J. R. , Goldmuntz E. , Chung W. K. , Seidman J. G. , Gelb B. D. , Seidman C. E. , Lifton R. P. , and Brueckner M. , Contribution of Rare Inherited and De Novo Variants in 2,871 Congenital Heart Disease Probands, Nature Genetics. (2017) 49, 1593–1601, 10.1038/ng.3970, 2-s2.0-85032451853, 28991257. 28991257 PMC5675000

[28] Kodo K. , Nishizawa T. , Furutani M. , Arai S. , Ishihara K. , Oda M. , Makino S. , Fukuda K. , Takahashi T. , Matsuoka R. , Nakanishi T. , and Yamagishi H. , Genetic Analysis of Essential Cardiac Transcription Factors in 256 Patients With Non-Syndromic Congenital Heart Defects, Circulation Journal. (2012) 76, no. 7, 1703–1711, 10.1253/circj.CJ-11-1389, 2-s2.0-84863207894, 22498567. 22498567

[29] Vasicek R. , Meinhardt G. , Haidweger E. , Rotheneder H. , Husslein P. , and Knöfler M. , Expression of the Human Hand1 Gene in Trophoblastic Cells Is Transcriptionally Regulated by Activating and Repressing Specificity Protein (Sp)-Elements, Gene. (2003) 302, no. 1-2, 115–127, 10.1016/S0378-1119(02)01096-X, 2-s2.0-0037413704, 12527202. 12527202

